# Porcine epidemic diarrhea virus (PEDV) detection and antibody response in commercial growing pigs

**DOI:** 10.1186/s12917-016-0725-5

**Published:** 2016-06-10

**Authors:** Jordan Bjustrom-Kraft, Katie Woodard, Luis Giménez-Lirola, Marisa Rotolo, Chong Wang, Yaxuan Sun, Peter Lasley, Jianqiang Zhang, David Baum, Phillip Gauger, Rodger Main, Jeffrey Zimmerman

**Affiliations:** Department of Veterinary Diagnostic and Production Animal Medicine, College of Veterinary Medicine, Iowa State University, 1850 Christensen Drive, Ames, IA 50011-1134 USA; Department of Statistics, College of Liberal Arts and Sciences, Iowa State University, Osborn Drive, Ames, IA 50011 USA; Smithfield Hog Production Missouri, 17999 US Highway 65, Princeton, MO 64673 USA

**Keywords:** PEDV, Virus shedding, Antibody kinetics, Oral fluids, Surveillance, IgG, IgA

## Abstract

**Background:**

Longitudinal samples from two production sites were used to (1) describe the pattern of PEDV shedding (rRT-PCR) in individual rectal swabs, pen fecal samples, and pen oral fluids (OF); (2) describe the kinetics of PEDV antibody by ELISA (IgA, IgG) testing of pig serum and pen oral fluid samples; and (3) establish cutoffs and performance estimates for PEDV WV ELISAs (IgA, IgG). Site One was PEDV positive; Site Two was PEDV negative. On Site One, pen samples (feces and oral fluids) and pig samples (rectal swabs and sera) were collected both before and after the population was exposed to PEDV.

**Results:**

On Site Two, pen oral fluid samples and individual pig serum samples were negative for both PEDV antibody and nucleic acid. On Site One, PEDV was detected by rRT-PCR at 6 days post exposure (DPE) in all sample types. The last rRT-PCR positives were detected in rectal swabs and oral fluids on 69 DPE. IgG and IgA were detected in oral fluids and serum samples by 13 DPE. Analysis of the PEDV serum IgG WV ELISA data showed that a sample-to-positive (S/P) cutoff of ≥ 0.80 provided a diagnostic sensitivity of 0.87 (95 % CI: 0.82, 0.91) and specificity of 0.99 (95 % CI: 0.98, 1.00). Serum IgG results declined slowly over the monitoring period, with 60 % of serum samples positive (S/P ≥ 0.80) at the final sampling on 111 DPE. Analysis of the PEDV oral fluid IgA WV ELISA found that a cutoff of S/P ≥ 0.80 provided a diagnostic sensitivity of 1.00 (95 % CI: 0.92, 1.00) and a diagnostic specificity of 1.00 (95 % CI: 0.99, 1.00). The oral fluid IgA response increased through 96 DPE and began to decline at the last sampling on 111 DPE.

**Conclusions:**

This study showed that oral fluid-based testing could provide an easy and “animal-friendly” approach to sample collection for nucleic acid and/or antibody-based surveillance of PEDV in swine populations.

## Background

Porcine epidemic diarrhea virus (PEDV) is an enveloped, single-stranded, positive-sense RNA virus in the family *Coronaviridae* [1]. In susceptible herds, PEDV is characterized by the rapid onset of watery diarrhea and vomiting in pigs of all ages, with mortality approaching 100 % in suckling piglets. First identified in 1978, PEDV was not considered a serious threat to swine health until devastating outbreaks of PEDV were reported in China in 2006 in association with previously unrecognized genetic variants [2]. Thereafter, pathogenic strains producing clinical PED outbreaks were reported in Japan, Korea, Thailand, the Philippines, the Western hemisphere and subsequently Portugal and Germany [3–5]. Thus, in a relatively short time, pathogenic PEDV has become pandemic.

Since there is little possibility that PEDV will soon be eradicated, it is important to identify the means to prevent and/or control its effects: PEDV management will necessitate monitoring PEDV in swine populations. Therefore, the purpose of this study was to (1) describe the patterns of PEDV shedding and detection in growing pigs as shown by PEDV real-time reverse transcription polymerase chain reaction (rRT-PCR) testing of individual pig rectal swabs, pen fecal samples, and pen oral fluids; (2) describe PEDV antibody kinetics as shown by enzyme-linked immunosorbent assay (ELISA) detection of IgA and IgG in individual pig serum and pen oral fluid samples; and (3) estimate the cutoffs and performance of the PEDV "whole virus" IgA and IgG ELISAs (WV IgA or IgG ELISA).

## Methods

### Experimental design

Individual pig samples (rectal swabs and/or serum) and pen samples (fecal and/or oral fluid specimens) were collected longitudinally from one PEDV-positive commercial wean-to-finish (WTF) barn in Missouri USA (Site One) and one PEDV-negative commercial WTF barn in Iowa USA (Site Two). Fecal samples and oral fluids were tested by PEDV real-time reverse-transcriptase polymerase chain reaction (rRT-PCR). Serum and oral fluid specimens were tested by two PEDV antibody WV ELISAs (IgA, IgG). Testing results were used to describe PEDV shedding, establish the performance parameters of two PEDV WV ELISAs (IgA, IgG), and characterize antibody kinetics in a commercial pig production system. This project was approved in writing both by an agent representing the livestock producer and the Iowa State University Office for Responsible Research.

### Site descriptions

Site One was a 52-pen WTF barn stocked with ~800 pigs. Pens were separated by metal gates, with 26 pens on each side of the walk way. Pens were equipped with automatic feeders, bowl drinkers, and fully slatted floors. The facility was designed with negative pressure tunnel ventilation and a deep pit (2.4 m) manure handling system. Pigs were placed in the facility at the time of weaning (~3 weeks of age). Pen samples (feces and oral fluids) and pig samples (rectal swabs and serum) were collected from the same 6 pens and a convenience sample of 5 pigs in each of the 6 pens at each sampling point. Sampling began when the pigs were ~3 weeks of age and continued at ~2-week intervals for 27 weeks. At 10 weeks post-placement, i.e., when pigs were approximately 13 weeks of age, the producer exposed the pigs (replacement gilts) to PEDV by mixing PEDV-positive fecal material with water and spraying feed and the pigs’ oral-nasal area with the mixture using a hand-held sprayer.

Site Two consisted of 3 identical 40-pen WTF barns, each stocked with ~900 pigs. Pens were separated by solid walls, with 20 pens on each side of the walk way. Pens were equipped with automatic feeders, bowl drinkers, and half-slatted floors. The barns were constructed with natural ventilation and deep pit (2.4 m) manure handling systems. Pigs were placed in the facility at the time of weaning (~3 weeks of age). Pen oral fluid samples were collected from 36 pens (4 pens were not stocked) in each of the 3 barns and serum samples were collected from a convenience sample of 20 pigs in 2 pens (10 pigs per pen) in each barn. Sampling began at two weeks post placement (pigs were ~5 weeks of age) and continued weekly for a total of 9 samplings. Individual pig rectal swabs and pen fecal specimens were not collected on Site Two.

### Sample collection

Individual pigs were restrained and bled using 12.5 mL vacutainer tubes (Covidien, Minneapolis, MN USA) and 20 gauge x 3.81 cm (1 ½ in.) needles (Smiths Medical, Dublin, OH USA). Blood samples were centrifuged at the laboratory, aliquoted, and stored at −20 °C.

Fecal swabs were collected from individual pigs using a commercial collection and transport system (StarswabII®, Starplex® Scientific Inc., Cleveland, TN USA) and stored at −20 °C. Prior to testing, swabs were suspended in 1 mL of PBS (1X pH 7.4, Invitrogen Corporation, Carlsbad, CA USA), vortexed, and the liquid submitted for testing by PEDV rRT-PCR.

Each pen-level fecal sample consisted of a convenience sample of 3-to-5 fresh semi-solid feces from throughout the pen. Approximately equal portions of pen feces were placed in one 50 mL tube (Thermo Fisher Scientific, Waltham, MA USA) and stored at −20 °C. Prior to testing, samples were homogenized (stirred), ~1.0 g placed in 1 mL of PBS (1X pH 7.4, Invitrogen Corporation) and submitted for PEDV rRT-PCR testing.

Pen-based oral fluids were collected as described elsewhere [6]. In brief, 3-strand, 100 % cotton rope was cut with the free end at shoulder height to the animals and suspended in the pen for 20 to 30 min. Pigs actively sought out and chewed the rope, leaving the strands moistened with oral fluids. The rope was then removed from the pen and the wet portion placed in a single-use plastic bag. Oral fluids were extracted by either manual or mechanical compression (wringer) of the wet rope, after which the fluid was decanted into 50 mL centrifuge tubes (Fisher Scientific) and stored at −20 °C.

For each site, all samples were completely randomized (random.org) within specimen type and submitted for testing at the end of the collection period.

### Diagnostic testing

#### PEDV RNA extraction and real-time reverse transcriptase PCR (rRT-PCR)

In brief, 90 μl of viral RNA was eluted from rectal swabs, fecal samples and oral fluid specimens using the Ambion® MagMAX™ viral RNA isolation kit (Life Technologies, Carlsbad CA USA) and a KingFisher® 96 magnetic particle processor (Thermo-Fisher Scientific) following the procedures provided by the manufacturers. Samples were tested for PEDV using a PEDV N gene-based rRT-PCR described in Madson et al. [7] and performed routinely at the Iowa State University-Veterinary Diagnostic Laboratory (ISU-VDL SOP 9.5263). The forward primer sequence was 5′-CGCAAAGACTGAACCCACTAACCT-3′, the reverse primer sequence was 5′-TTGCCTCTGTTGTTACTTGGAGAT-3′, and probe sequence was 5′-FAM-TGTTGCCAT/ZEN/TACCACGACTCCTGC-Iowa Black-3′. The eluted RNA, primers, and probe were mixed with commercial reagents TaqMan® Fast Virus 1-Step Master Mix (Life Technologies) and the rRT-PCR reactions were conducted on an ABI 7500 Fast instrument (Life Technologies) in fast mode as follows: 1 cycle at 50 °C for 5 min, 1 cycle at 95 °C for 20 s, 40 cycles at 95 °C for 3 s, and 60 °C for 30 s. The results were analyzed using an automatic baseline setting with a threshold at 0.1. Quantification cycle (Cq) values < 35 were considered positive for the corresponding coronavirus. Data were reported as ‘adjusted Cqs’:

1 $$ \mathrm{Adjusted}\ \mathrm{C}\mathrm{q} = \left(35\ \hbox{--}\ \mathrm{sample}\ \mathrm{C}\mathrm{q}\right) $$

#### PEDV whole virus (WV) antibody ELISA

A U.S. prototype PEDV isolate (USA/NC35140/2013, [8]) was used in the PEDV WV antibody ELISA. Each batch of approximately 1000 mL of PEDV was propagated on Vero cells (ATCC CCL-81). Briefly, one 75 cm^2^ flask (Thermo Fisher Scientific) of confluent Vero cells was inoculated with 3 mL of PEDV stock (1x10^5^ TCID_50_ per mL) followed by the addition of 50 mL of cell culture medium composed of MEM 1X (Minimum Essential Medium, Life Technologies) supplemented with 0.3 % tryptose phosphate broth, 0.02 % yeast extract, 5 ug per mL Trypsin 250 (Sigma-Aldrich, St. Louis, MO USA), plus penicillin/streptomycin (10 U per mL), gentamicin (0.05 mg per mL) and amphotericin (0.25 μg per mL) as antibiotics. After 3 to 4 days at 37 °C in a 5 % CO_2_ incubator and when cytopathic effects were apparent, the contents of the flask (53 mL) were used to further expand the virus by inoculating each of 4 875 cm^2^ flasks (BD Falcon, San Jose, CA) containing confluent Vero cell monolayers with 13 mL of the harvested PEDV plus 240 mL of culture medium. After 3 to 4 days of incubation and when cytopathic effects were apparent, the fluid was frozen (−80 °C), thawed, poured off, and then centrifuged at 4,000 x *g* for 15 min to remove cell debris. The virus was pelleted by ultracentrifugation at 140,992 x *g* for 3 h, after which the pellet was washed twice with sterile PBS (1X pH 7.4) to remove culture medium components. The purified virus was re-suspended in 100µl PBS (1X pH 7.4) at a 1:100 dilution of the original supernatant volume and stored at −80 °C. Following titration and optimal dilution (PBS pH 7.4), polystyrene 96-well microtitration plates (Nalge Nunc, Rochester, NY USA) were manually coated (100 μl per well) with the viral antigen solution and incubated at 4 °C overnight. After incubation, plates were washed 5 times, blocked with 300 μl per well of a solution containing 1 % bovine serum albumin (Jackson ImmunoResearch Inc., West Grove, PA USA), and incubated at 25 °C for 2 h. Plates were then dried at 37 °C for 4 h and stored at 4 °C in a sealed bag with desiccant packs. The performance of each lot of plates was standardized using a panel of reference PEDV negatives and positives. Plate lots with a coefficient of variation ≥10 % were rejected.

ELISA conditions for the detection of anti-PEDV IgA and IgG antibodies in serum and oral fluid specimens, including coating and blocking conditions, reagent concentrations, incubation times, and buffers, were identical. Serum samples were diluted 1:50 and oral fluid samples were diluted 1:2, after which plates were loaded with 100 μl of the diluted sample per well. Plates were incubated at 25 °C (serum) or 37 °C (oral fluid) for 1 h and then washed 5 times with PBS (1X pH 7.4). Positive and negative plate controls, i.e., antibody-positive and -negative experimental serum samples, were run in duplicate on each ELISA plate.

To perform the assay, 100 μl of peroxidase-conjugated goat anti-pig IgG (Fc) antibody (Bethyl Laboratories Inc., Montgomery, TX USA) diluted 1:20,000 for serum and 1:3,000 for oral fluid samples or goat anti-pig IgA (Bethyl Laboratories Inc.) diluted 1:7,000 for serum and 1:3,000 for oral fluid samples was added to each well and the plates incubated at 25 °C (serum) or 37 °C (oral fluid) for 1 h. After a washing step, the reaction was visualized by adding 100 μl of tetramethylbenzidine-hydrogen peroxide (Dako North America, Inc., Carpinteria, CA USA) substrate solution to each well. After 5 min incubation at room temperature, the reaction was stopped by the addition of 50 μl of stop solution (1 M sulfuric acid) to each well. Reactions were measured as optical density (OD) at 450 nm using an ELISA plate reader (Biotek^®^ Instruments Inc., Winooski, VT USA) operated with commercial software (GEN5™, Biotek^®^ Instruments Inc.). The antibody response in serum and oral fluid samples was represented as sample-to-positive (S/P) ratios calculated as:

2 $$ \mathrm{S}/\mathrm{P}\ \mathrm{ratio} = \frac{\left(\mathrm{sample}\ \mathrm{O}\mathrm{D}\ \hbox{--}\ \mathrm{negative}\ \mathrm{control}\ \mathrm{mean}\ \mathrm{O}\mathrm{D}\right)}{\left(\mathrm{positive}\ \mathrm{control}\ \mathrm{mean}\ \mathrm{O}\mathrm{D}\ \hbox{--}\ \mathrm{negative}\ \mathrm{control}\ \mathrm{mean}\ \mathrm{O}\mathrm{D}\right)} $$

### Data analysis

Statistical analyses were performed using commercial statistical software (SAS® Version 9.4, SAS® Institute, Inc., Cary, NC) using test results on serum (Site One, *n* = 330; Site Two, *n* = 540), oral fluid (Site One, *n* = 66; Site 2, *n* = 972), rectal swabs (Site One, *n* = 330), and pen feces (Site One, *n* = 66). A mixed-effects repeated measures model (Proc GLIMMIX) was used to analyze the association between the detection of PEDV by rRT-PCR and the variables of interest, i.e., sample specimen (oral fluids, rectal swab, pen feces, serum) and day post exposure (DPE) using pen as a random effect. Fixed effects were considered significant at α = 0.05. Differences in the proportion of PEDV rRT-PCR positive oral fluid, rectal swab, and pen feces was compared using the Fisher Exact Test. Point and interval estimates of the sensitivity and specificity of the PEDV WV IgG and IgA ELISAs for serum and oral fluid samples were calculated using the exact Binomial formula and confidence intervals.

## Results

### PEDV rRT-PCR

On Site Two, all oral fluid samples (*n* = 972) collected during the monitoring period were PEDV rRT-PCR-negative.

**Fig. 1:**
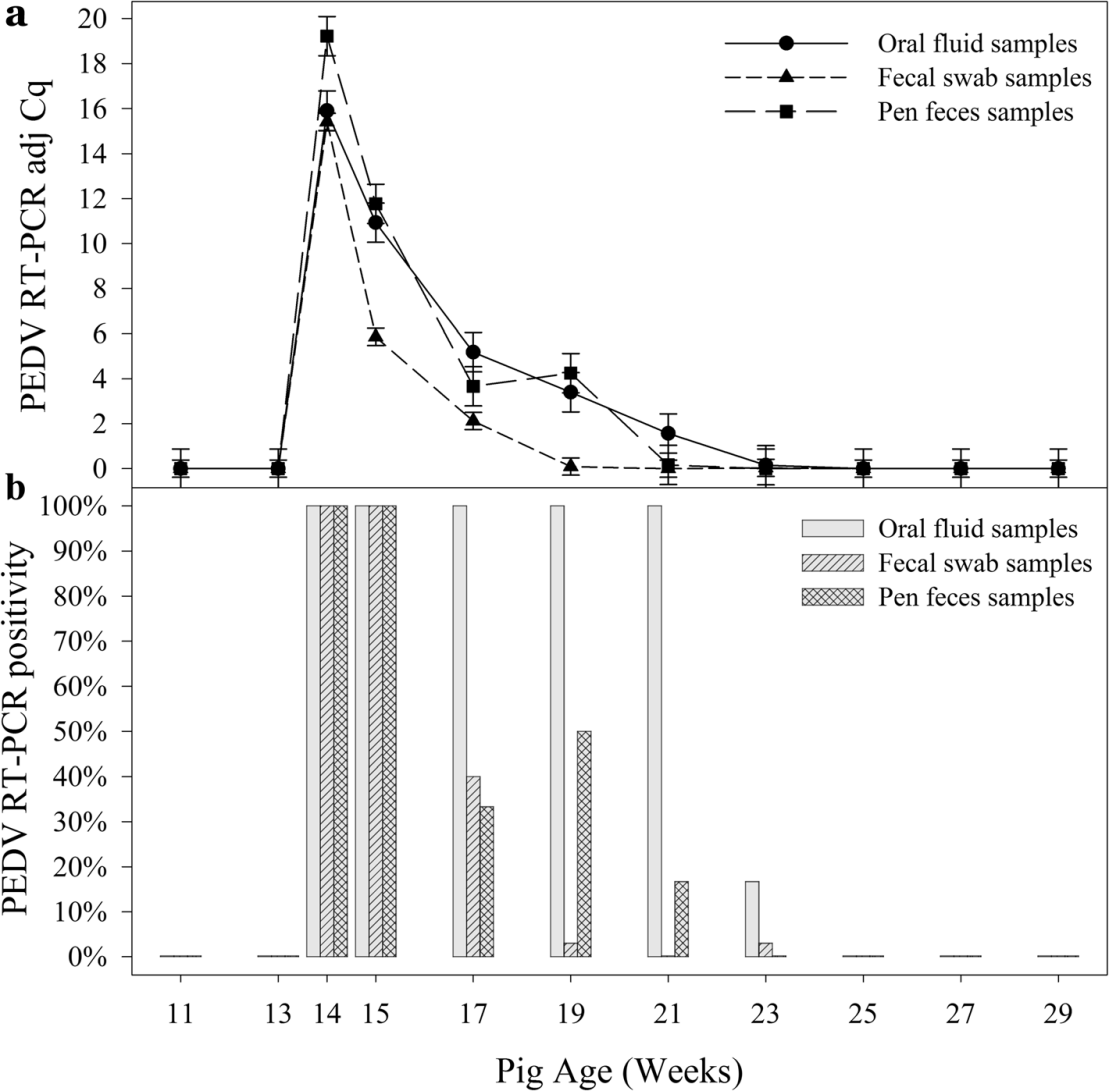
Detection of porcine epidemic diarrhea virus (PEDV) in pig rectal swabs, pen-based oral fluids, and pen-based fecal specimens from Site One by rRT-PCR. At 13 weeks of age, the producer exposed the pigs to PEDV-positive fecal material mixed with water using a hand-held sprayer. **a** (above): Mean adjusted quantification cycle (Cq) (35 – sample Cq) of positive samples. **b** (below): Proportion of positive samples

On Site One, PEDV was detected in individual rectal swabs, pen fecal samples, and pen oral fluids by rRT-PCR collected for 10 weeks post exposure (Fig. 1a), i.e., through 69 DPE (23 weeks of age). An analysis of the adjusted rRT-PCR Cq values showed differences in the concentration of PEDV in the three sample types over time (*p* = 0.0005). The concentration of PEDV was higher in pen fecal samples compared to rectal swabs (*p* = 0.0001) and oral fluids (*p* = 0.0088) at 6 DPE. Thereafter, no difference was detected in the concentration of virus in oral fluid and pen fecal samples through 69 DPE. In contrast, the concentration of PEDV in rectal swab samples was significantly lower than in pen fecal samples and oral fluid samples at 13, 27, and 41 DPE (15, 17, 19 weeks of age) (*p* < 0.002).

An analysis of the proportion of rRT-PCR positive samples (Fig. 1b) found differences among specimen types at 27, 41, and 55 DPE (17, 19, 21 weeks of age) (Fisher’s Exact Test, *p* < 0.03). No differences were found between pen fecal samples and oral fluid samples over the monitoring period, except at 55 DPE when 6 of 6 oral fluid and 1 of 6 pen fecal samples were positive (*p* = 0.015). However, the proportion of positive oral fluid specimens was significantly greater than rectal swabs at 27, 41, and 55 DPE (17, 19, 21 weeks of age) (*p* < 0.02). Likewise, the proportion of positive pen fecal samples at 41 DPE was significantly greater than rectal swabs (*p* = 0.0012).

### PEDV whole virus (WV) antibody ELISA

**Fig. 2:**
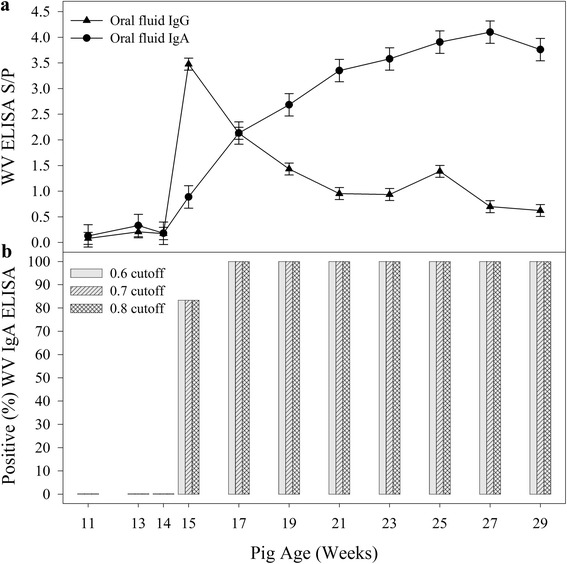
PEDV Whole Virus ELISA IgG and IgA responses in oral fluid samples following exposure to PEDV at 13 weeks of age. **a** (above): Oral fluid IgG and IgA responses over time. **b** (below): Proportion of positive oral fluid IgA samples at three different S/P cutoffs

**Fig. 3:**
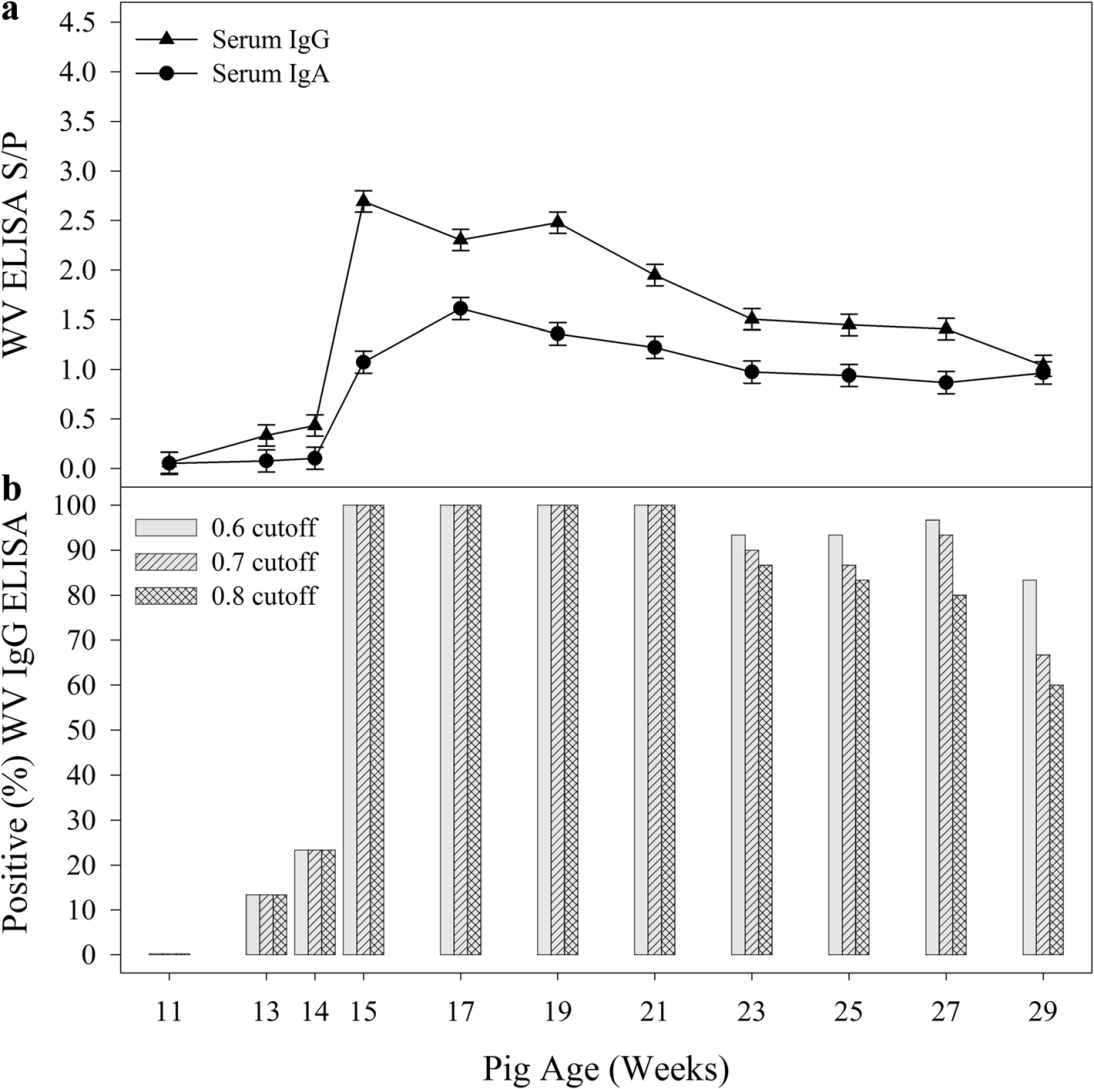
PEDV Whole Virus ELISA IgG and IgA response in serum samples following exposure to PEDV at 13 weeks of age. **a** (above): Serum IgG and IgA responses over time. **b** (below): Proportion of positive serum IgG samples at three different S/P cutoffs

On Site One, PEDV IgG and IgA were detected in all oral fluid and serum samples collected after 13 DPE (≥15 weeks of age). As shown in Figs. 2a and 3a, the oral fluid IgA S/P responses increased until 97 DPE (27 weeks of age), whereas the serum IgA response peaked at 27 DPE (17 weeks of age). Figures 2b and 3b show the percent positive oral fluid samples and serum samples, respectively, for three S/P cutoffs. Oral fluid (*n* = 972) and serum samples (*n* = 540) from Site Two were used as a source of PEDV negative samples for calculating cutoffs and performance estimates for the PEDV WV IgA and IgG ELISAs using the exact Binomial formula and confidence intervals (Table 1).

**Table 1:** Porcine epidemic diarrhea virus (PEDV) whole virus (WV) ELISA sensitivity (Se) and specificity (Sp) by specimen type, antibody isotype, and sample-to-positive (S/P) ratio^a^

Oral fluid - PEDV WV IgG ELISA			Oral fluid - PEDV WV IgA ELISA		
S/P	Se (95 % CI)	Sp (95 % CI)	S/P	Se (95 % CI)	Sp (95 % CI)
** 0.30**	1.00 (0.92, 1.00)	0.86 (0.84, 0.89)	**0.30**	1.00 (0.92, 1.00)	0.88 (0.86, 0.90)
**0.40**	1.00 (0.92, 1.00)	0.90 (0.88, 0.92)	**0.40**	1.00 (0.92, 1.00)	0.96 (0.94, 0.97)
**0.50**	0.98 (0.87, 1.00)	0.93 (0.91, 0.94)	**0.50**	1.00 (0.92, 1.00)	0.98 (0.97, 0.99)
**0.60**	0.88 (0.74, 0.96)	0.95 (0.93, 0.96)	**0.60**	1.00 (0.92, 1.00)	0.99 (0.99, 1.00)
**0.70**	0.71 (0.55, 0.84)	0.96 (0.95, 0.97)	**0.70**	1.00 (0.92, 1.00)	1.00 (0.99, 1.00)
**0.80**	0.69 (0.53, 0.82)	0.97 (0.96, 0.98)	**0.80**	1.00 (0.92, 1.00)	1.00 (0.99, 1.00)
Serum - PEDV WV IgG ELISA			Serum - PEDV WV IgA ELISA		
S/P	Se (95 % CI)	Sp (95 % CI)	S/P	Se (95 % CI)	Sp (95 % CI)
**0.30**	1.00 (0.98, 1.00)	0.95 (0.93, 0.96)	**0.30**	0.93 (0.88, 0.96)	1.00 (0.99, 1.00)
**0.40**	0.99 (0.96, 1.00)	0.98 (0.97, 0.99)	**0.40**	0.90 (0.86, 0.94)	1.00 (0.99, 1.00)
**0.50**	0.97 (0.93, 0.99)	0.99 (0.98, 1.00)	**0.50**	0.82 (0.76, 0.87)	1.00 (0.99, 1.00)
**0.60**	0.95 (0.91, 0.86)	0.99 (0.98, 1.00)	**0.60**	0.73 (0.67, 0.79)	1.00 (0.99, 1.00)
**0.70**	0.91 (0.86, 0.94)	0.99 (0.98, 1.00)	**0.70**	0.66 (0.59, 0.72)	1.00 (0.99, 1.00)
**0.80**	0.87 (0.82, 0.91)	0.99 (0.98, 1.00)	**0.80**	0.58 (0.51, 0.65)	1.00 (0.99, 1.00)

^a^ $$ \mathrm{S}/\mathrm{P}=\frac{\left(\mathrm{sample}\ \mathrm{O}\mathrm{D}\ \hbox{--}\ \mathrm{negative}\ \mathrm{control}\ \mathrm{mean}\ \mathrm{O}\mathrm{D}\right)}{\left(\mathrm{positive}\ \mathrm{control}\ \mathrm{mean}\ \mathrm{O}\mathrm{D}\ \hbox{--}\ \mathrm{negative}\ \mathrm{control}\ \mathrm{mean}\ \mathrm{O}\mathrm{D}\right)} $$

## Discussion

The first objective of this study was to compare the detection of PEDV by rRT-PCR in rectal swabs, pen fecal samples, and oral fluid samples from pigs housed in commercial WTF facilities. Specifically, comparisons were made among specimens in the duration of PEDV detection, proportion of positive samples, and concentration of virus in positive samples.

PEDV was detected by rRT-PCR in rectal swabs, pen fecal samples, and oral fluid samples, with the last rRT-PCR positive rectal swabs collected at 69 DPE, pen fecal samples at 55 DPE, and oral fluid samples at 69 DPE. The fact that the cessation of PEDV detection coincided in fecal and oral fluid samples suggested that the environment did not serve as a reservoir for PEDV. Previous publications provided data with which rectal swab data could be compared, but a comprehensive search of the literature did not find previous reports on the detection of PEDV in pen feces or pen-based oral fluid samples. Madson et al. [7] detected PEDV in rectal swabs through 24 days post inoculation (DPI) in 5 of 8 pigs inoculated at 3 weeks of age with PEDV isolate US/Iowa/18984/2013. Thomas et al. [9] detected PEDV in rectal swabs for up to 21 DPI in 3-week-old pigs inoculated with PEDV isolate US/IN19338/2013. Crawford et al. [10] detected PEDV in rectal swabs for up to 42 DPI in 4-week-old pigs infected by contact with a pig inoculated with PEDV isolate US/Colorado/2013.

The concentration of virus, as measured by rRT-PCR, differed among specimen types. In particular, the concentration of PEDV nucleic acid in individual pig rectal swabs was significantly lower than oral fluid or pen-based fecal samples. The concentration of virus in PEDV rRT-PCR-positive oral fluid and pen-based fecal samples was not significantly different, except at 6 DPE.

Differences were also detected among specimen types in the proportion of positive samples by time. All oral fluid samples were rRT-PCR positive (6 of 6) through 55 DPE while the number PEDV rRT-PCR-positive pen fecal samples and rectal swabs declined to ≤ 50 % at 27 DPE and later. The lower concentration and lower rate of detection in rectal swabs could be attributed to the small volume of sample retained by the swab plus the effect of diluting each rectal swab in one mL of PBS prior to testing. The lower rate of detection in pen floor fecal samples may reflect the non-uniform distribution of positive samples within a pen. Previously, O’Connor et al. [11] reported differences in *Salmonella* concentrations at various locations within a pen, i.e., the distribution of *Salmonella* within a pen was not uniform.

Detection of PEDV by rRT-PCR using pen-based oral fluid samples has not previously been reported in the refereed literature. Using the described procedures, one oral fluid sample from a pen provided detection equal to, or better than, rectal swab samples from 5 pigs in the pen. Likewise, detection using oral fluid samples was equal to, or better than, detection using pen fecal samples. Thus, the data indicated that oral fluids were an effective and sensitive specimen for herd-level rRT-PCR-based detection of PEDV in commercial growing pig environments.

The second objective of the study was to describe PEDV serum and oral fluid IgA and IgG antibody kinetics and to estimate the performance of the PEDV “whole virus” IgA and IgG indirect ELISAs at different cutoffs.

For serum IgG and IgA, respectively, a cutoff of S/P ≥ 0.80 provided diagnostic sensitivities of 0.87 (95 % CI: 0.82, 0.91) and 0.58 (95 % CI: 0.51, 0.65) and diagnostic specificities of 0.99 (95 % CI: 0.98, 1.00) and 1.00 (95 % CI: 0.99, 1.00). Although both serum IgG and serum IgA were detected by 13 DPE, the serum IgG response provided better diagnostic performance than serum IgA (Table 1). Serum IgG results declined slowly over the monitoring period, with 60 % of serum samples positive (S/P ≥ 0.80) at the final sampling on 111 DPE. The utility of the PEDV WV serum IgA ELISA is a question for future research. In particular, research is needed to determine whether the detection of serum IgA, i.e., an antibody isotype necessarily produced by the piglet in response to infection, could be used to identify infection in the face of PEDV-specific colostral (IgG) antibody or whether serum IgA response could be used  in a confirmatory assay to clarify equivocal PEDV WV IgG ELISA results.

For oral fluid IgG and IgA, respectively, a cutoff of S/P ≥ 0.80 provided diagnostic sensitivities of 0.69 (95 % CI: 0.53, 0.82) and 1.00 (95 % CI: 0.92, 1.00) and diagnostic specificities of 0.97 % (95 % CI: 0.96, 0.98) and 1.00 (95 % CI: 0.99, 1.00). Although oral fluid IgG and IgA were detected by 13 DPE, the oral fluid IgA response gave better diagnostic performance than IgG. Notably, the oral fluid IgA response increased through 96 DPE and only began to decline at the last sampling on 111 DPE.

There are no prior reports against which to directly compare the PEDV oral fluid antibody kinetics observed in the current study, but DeBuysscher and Berman [12] reported a large increase in IgA-secreting cells within the salivary glands of pigs following oral exposure to another coronavirus, transmissible gastroenteritis virus (TGEV). On the other hand, Brandtzæg [13] noted that enteric stimulation does not necessarily produce a strong salivary IgA response in humans. Because of similarities in experimental design, these data may also be compared to oral fluid IgG and IgA responses reported for porcine reproductive and respiratory syndrome virus (PRRSV) and influenza A virus (IAV) [14, 15]. Kittawornrat et al. [14] evaluated PRRSV oral fluid IgG and IgA responses using pen-based field samples and experimental oral fluid samples. Using a commercial PRRS serum antibody ELISA adapted to oral fluids, IgG was readily detected and provided a diagnostic sensitivity of 0.95 (95 % CI: 0.92, 0.97) and specificity of 1.00 (95 % CI: 0.99, 1.00). In contrast, the IgA response in oral fluid was detectable, but weak and transient. Panyasing et al. [15] evaluated influenza A virus IgG and IgA responses in oral fluids. Unlike the PRRSV response, both anti-IAV IgG and IgA were readily detected in oral fluids by ~7 DPI and throughout the study (DPI 42). These studies suggest that oral fluid IgG and IgA kinetics vary among pathogens. Thus, it will be critical to evaluate antibody isotype kinetics during the process of adapting antibody assays to the swine oral fluid matrix.

For disease surveillance in swine populations, diagnostic specificity is paramount because false positives quickly erode confidence in test results. Therefore, the investigators recommend a conservative S/P cutoff for serum and oral fluid samples, e.g., ≥ 0.80 for routine use. However, diagnostic sensitivity and specificity were presented for several PEDV WV ELISA S/P cutoffs (Table 1) to allow users to interpret results in the context of specific circumstances.

## Conclusion

The purpose of surveillance is to provide timely information on pathogen exposure and immune responses in swine populations in order to optimize health and prevent disease. Well-validated, reproducible, high-throughput nucleic acid and antibody assays are necessary to achieve this purpose. This study showed that oral fluid-based testing could provide an easy and “animal-friendly” approach to nucleic acid and/or antibody-based surveillance of PEDV in swine populations. In particular, the exceptional strength and duration of the PEDV IgA antibody response in oral fluids raises the question as to its ability to serve as an indicator of protective immunity; this is a question for future research.

## Abbreviations

CI, confidence interval; DPE, day post exposure; DPI, day post inoculation; ELISA, enzyme-linked immunosorbent assay; IAV, influenza A virus; IgA, immunoglobulin A; IgG, immunoglobulin G; ISU-VDL, Iowa State University Veterinary Diagnostic Laboratory; OD, optical density; PBS, phosphate-buffered saline; PED/PEDV, porcine epidemic diarrhea/PED Virus; PRRS/PRRSV, porcine reproductive and respiratory syndrome/PRRS virus; rRT-PCR, real-time reverse transcription polymerase chain reaction; S/P, sample-to-positive; TGEV, transmissible gastroenteritis virus; WTF, wean-to-finish; WV ELISA, whole virus enzyme-linked immunosorbent assay
